# Classification of Wheelchair Related Shoulder Loading Activities from Wearable Sensor Data: A Machine Learning Approach

**DOI:** 10.3390/s22197404

**Published:** 2022-09-29

**Authors:** Wiebe H. K. de Vries, Sabrina Amrein, Ursina Arnet, Laura Mayrhuber, Cristina Ehrmann, H. E. J. Veeger

**Affiliations:** 1 Swiss Paraplegic Research, Guido A. Zachstrasse 4, 6207 Nottwil, Switzerland; 2 Rehabilitation Engineering Laboratory, Hönggerberg Campus, ETH Zurich, 8049 Zurich, Switzerland; 3 Department of Biomechanical Engineering, Delft University of Technology, Mekelweg 2, 2628 CD Delft, The Netherlands

**Keywords:** wheelchair, shoulder loading activities, wearable sensors, deep learning, classification

## Abstract

Shoulder problems (pain and pathology) are highly prevalent in manual wheelchair users with spinal cord injury. These problems lead to limitations in activities of daily life (ADL), labor- and leisure participation, and increase the health care costs. Shoulder problems are often associated with the long-term reliance on the upper limbs, and the accompanying “shoulder load”. To make an estimation of daily shoulder load, it is crucial to know which ADL are performed and how these are executed in the free-living environment (in terms of magnitude, frequency, and duration). The aim of this study was to develop and validate methodology for the classification of wheelchair related shoulder loading ADL (SL-ADL) from wearable sensor data. Ten able bodied participants equipped with five Shimmer sensors on a wheelchair and upper extremity performed eight relevant SL-ADL. Deep learning networks using bidirectional long short-term memory networks were trained on sensor data (acceleration, gyroscope signals and EMG), using video annotated activities as the target. Overall, the trained algorithm performed well, with an accuracy of 98% and specificity of 99%. When reducing the input for training the network to data from only one sensor, the overall performance decreased to around 80% for all performance measures. The use of only forearm sensor data led to a better performance than the use of the upper arm sensor data. It can be concluded that a generalizable algorithm could be trained by a deep learning network to classify wheelchair related SL-ADL from the wearable sensor data.

## 1. Introduction

### 1.1. Background

Shoulder problems such as pain, functional limitations, or damage of anatomical structures of the shoulder are highly prevalent in manual wheelchair users (MWU) with spinal cord injury (SCI) [1,2,3,4,5,6,7,8,9,10,11]. These problems lead to limitations in activities of daily life (ADL), labor- and leisure participation [10,11,12,13], increased costs due to pain medication, visits to the doctor and/or surgery, and reduced quality of life (QoL) [14].

Shoulder problems are often associated with the long-term reliance on the upper limbs [15] and the accompanying “shoulder load”. For instance, wheelchair related tasks such as weight relief lifts and transfers are activities performed multiple times per day, which result in a high load at the shoulder [16,17,18]. A wheelchair propulsion push results in a relatively low load on the shoulder, in terms of the magnitude of the joint contact forces [18], but due to the distance travelled [19] (and consequently high number of pushes), manual wheelchair propulsion might still result in a high exposure per day. 

If one, or a combination of the three factors, magnitude, frequency, and duration, which together define exposure, deviate from their ideal value, they become risk factors for overloading the shoulder joint and consequently the development of pain and pathology [20,21]. In the literature, there are numerous suggestions that shoulder load leads to shoulder problems [2,14,17,18,22,23,24,25,26,27,28], but there is only scattered evidence of such a relationship. Several cross-sectional studies have described the associations of single components of shoulder load (e.g., rate of rise of exerted force, direction of exerted force, propulsion pattern) with either pain or pathology [24,29,30,31,32,33].

In order to lower the high prevalence of shoulder problems in MWUs, individual recommendations on lowering shoulder load in daily life are needed. Although laboratory based measurements have been performed to investigate the shoulder load during certain wheelchair related activities such as wheelchair propulsion [18,34,35], weight relief lifts [17,24] or handcycling [35,36], no research has been conducted to investigate the actual loading of the shoulder on a daily basis and based on the activities MWUs perform. To make such an estimation of daily shoulder load in the population of MWUs with SCI, it is crucial to know *which* ADL is performed and *how* these ADL are executed in the free-living environment throughout the day (in terms of magnitude, frequency, and duration).

Wearable sensors such as inertial measurement units (IMUs) and electromyography (EMG) sensors are minimally intrusive measurement tools that can be used to quantify movements [37,38] and muscle activity over a longer period. The largest limitation of using IMUs lies in “integration drift” when fusing sensor signals into orientation estimates [39] and the temperature depending bias of gyroscopes has been reduced to a minimum in the more recent generations of sensors. Therefore, the use of only raw (or calibrated) wearable sensor data such as the acceleration and angular velocity for the classification of activities is a growing field of research, with promising results. Especially in the able-bodied population, IMUs or embedded sensors (e.g., smartphones, smartwatches) have been shown to be a valuable tool to monitor activities in a free-living environment [40,41,42], but only a few studies have investigated activity detection and classification among MWUs [37,43,44,45,46,47,48,49]. Previous research examining the use of wearable sensors has predominantly focused on physical activity detection to estimate the activity levels and energy expenditure in MWUs with SCI [50,51,52,53,54,55]. 

The machine learning techniques used in the detection and classifications of general activities in MWUs from wearable sensor data appear to have an overall accuracy of 90% and above [37,43,44,45,46,56]. Earlier approaches in activity monitoring in MWU used rule based algorithms to classify types of activity from sensor signals [47]. The drawback of such an approach becomes clear when expanding the list of activities to be classified: the set of rules has to be updated. Moreover, feature extraction of the sensor signals is by investigator design, which might become labor intensive or even problematic when scaling up to a larger number of activities to be classified. More recent developments in the deep learning domain overcome these limitations by automatic generation of optimal features from the raw input data [57]. Such deep learning algorithms could be a valid tool to classify specific shoulder loading activities of daily living (SL-ADL) in MWUs from wearable sensor data. 

Based on the duration and frequency of SL-ADL (measured in daily life) and the known magnitude of these activities (measured in the laboratory or taken from the literature [17,25,32]), an estimation of shoulder load experienced during the daily conditions can be made. As shoulder load in itself is a difficult variable to use as a target for future interventions (*“your shoulder load is too high”*), the detection of *which activities* are performed in terms of frequency and duration, as a proxy for shoulder load, is essential.

### 1.2. Aims and Contribution

This work tries to fill the existing gap in the knowledge and methodology for the monitoring of shoulder load in MWUs in real-life conditions. The overall objective of the current study was to develop and validate the methodology for the classification of wheelchair related SL-ADL based on wearable sensor data, with a preference for a generalizable algorithm. Generalizable means that once the algorithm is trained, it can be applied to new data from new participants, which are collected with the same setup, without additional training of the algorithm. We therefore aimed to determine the performance of the classifying algorithm by calculating its accuracy, sensitivity, precision, and specificity for a list of relevant wheelchair-related SL-ADL. In addition, we aimed to compare different combinations of sensors to identify the sensor setup with the best performance. We hypothesize that the setup with all five sensors will capture the most detail of the activities and result in the best classifying performance over all of SL-ADL, but that a setup with only three sensors (wheelchair frame and wheel, upper arm) will be sufficiently informative for classification of the SL-ADL of interest.

## 2. Materials and Methods

Ethical approval for this study was obtained from the Ethikkomission Nordwest- und Zentralschweiz (EKNZ), project-ID 2020-01961.

### 2.1. Participants

For the collection of data to develop the methodology, 10 able-bodied participants (seven females; age 39 ± 9.4 years; stature 169 ± 9.1 cm; weight 66 ± 12 kg) were invited for the study. After explanation of the project goal and format of the experiments, their informed consent was obtained. In the week before the actual measurements, all participants underwent training in the wheelchair related activities of interest until they were comfortable in a smooth execution of all activities of interest.

### 2.2. Instrumentation

**Figure 1:**
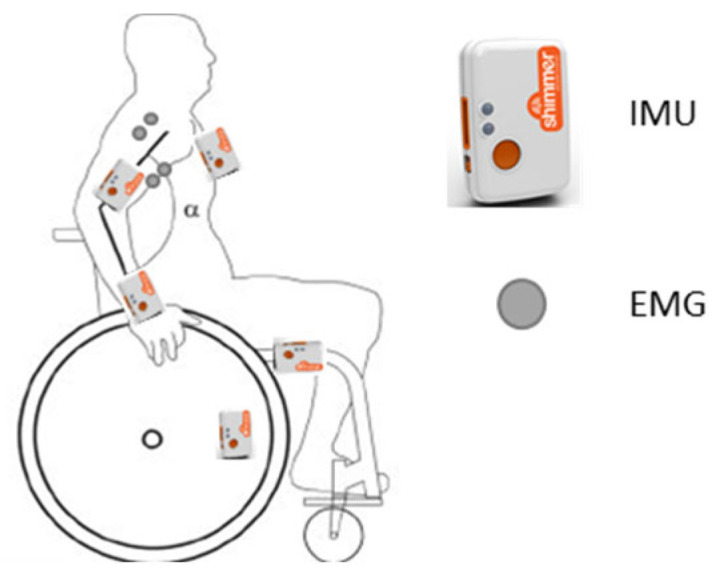
The instrumentation and placement.

To collect relevant data for the classification of wheelchair related shoulder loading activities, sensors were placed on those segments that actually move during these activities: wheelchair frame (WC) and wheel (WCW), thorax (Thor), right upper arm (UA), and forearm (FA). The sensors chosen were Shimmer IMUs sensors (Shimmer-3, Shimmer, Dublin, Ireland), a convenient and flexible wearable sensor platform for research purposes; however, any sensor platform that can measure acceleration and angular velocity with similar specifications should be applicable. The sensors on the upper and forearm were placed most distally for optimal measurement of the internal and external rotation and pro and supination, respectively [58]. The sensor at the upper arm additionally collected two channels of bipolar surface EMG of the long head of the biceps and the medial deltoid muscles (see also Figure 1). From the Shimmer data, acceleration and gyroscope signals were downsampled from 100 Hz to 10 Hz using a moving average filter; no further processing was applied. EMG signals were sampled at 1000 Hz using a 4th order bidirectional Butterworth filter consecutively high pass filtered at 20 Hz, offset corrected, rectified, and low pass filtered at 2 Hz to obtain a smooth envelope. This smooth rectified EMG was also downsampled to 10 Hz. Two static postures were measured to enable normalization of the collected EMG signals: (1) biceps brachialis long head: while actively sitting up straight, upper arm vertical along the thorax, elbow 90° flexed, both forearms parallel pointing frontal, thumbs up to ensure mid-position between pro- and supination, while holding a weight of 2 kg, and (2) medial deltoid: 90° of abduction, elbow stretched, thumbs pointing frontal, while holding a weight of 2 kg. 

A manual wheelchair (Küschall Compact, seat width 46 cm, 3° camber, Küschall AG, Witterswil, Switzerland) was used without further adaptation by the participants. All experimental trials were recorded on a consumer grade videorecorder (Canon Legria HF R806 HD, Canon, Switzerland) to label the collected data.

### 2.3. Experimental Trials

An initial list of the most relevant SL-ADL was extracted from the literature [13,14,16,17,25,37,56,59,60,61], and in discussion with clinicians from one of the specialized SCI centers in Switzerland (a medical doctor from the outpatient setting, a physiotherapist, and an occupational therapist), supplemented with several clinically relevant shoulder loading tasks. The resulting list of SL-ADLs that were addressed in this study is given in Table 1. After instruction, participants were free to perform the activities in their preferred fashion, except for the velocity on the treadmill. All activities were performed under laboratory conditions.

### 2.4. Video Annotation

Recorded video was annotated according the definitions from Table 1 using custom written MATLAB routines. Care was taken to indicate the start and end of a certain activity in the video recordings at the start of a movement that could also be recognized as such within the sensor signals to obtain a clear, consistent, and corresponding start and end point in both data streams (video and sensor) over all participants. 

**Figure 2:**
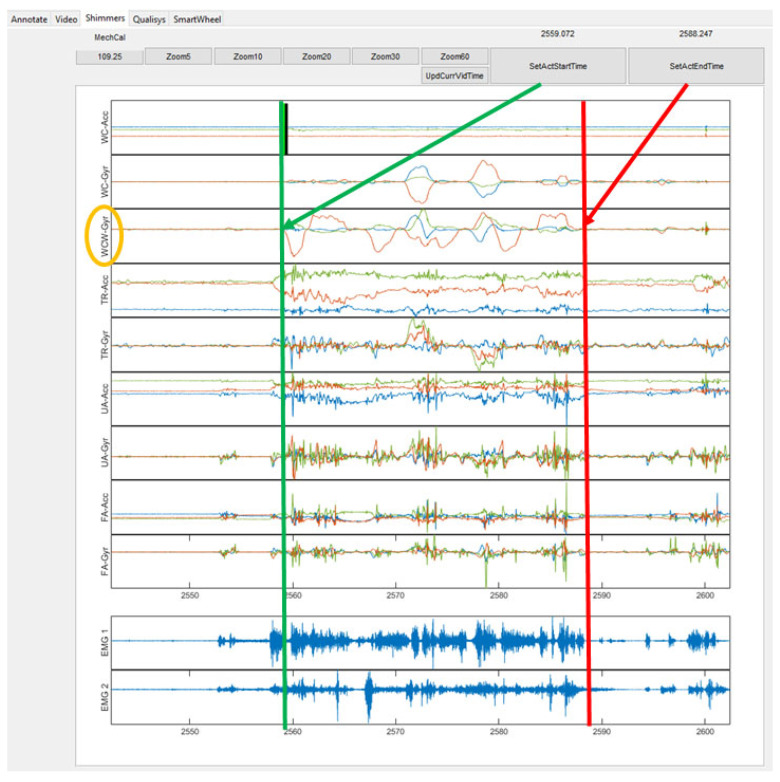
The video annotation of the start (green vertical line) and end (red vertical line) of the activity “Dribbling”; the gyroscope signal (yellow oval) from the wheel sensor was used to identify start and end of wheel rotation.

For example, as depicted in Figure 2, the start and end of the activity “Dribbling” can be clearly identified in the angular velocity signal of the wheelchair wheel, where the gyroscope signal from wheel sensor (WCW-Gyr) starts deviating from zero, or decreases to zero at standstill. For activities where no wheel movement was involved (manual material handling), the start of moving the hands toward the object to be manipulated could be easily detected in the acceleration and gyroscope signals from the forearm sensor. This procedure is important as any mismatch in the prediction (of onset and end) of activities with respect to the true class (activity as annotated in video) will be reflected in the measures used to quantify the performance of the algorithms (see Section 2.6).

### 2.5. Machine Learning and Deep Learning Techniques

The activities from the list of relevant SL-ADL are characterized as complex activities, composed of several sub-activities, with quite some variation in execution over the participants, and with varying duration. From the analysis of the pilot study data and literature [44,57,62], it became clear that for these complex activities, traditional machine learning classification techniques such as support vector machine (SVM), K-nearest neighbor (KNN), random forest, etc. require intense feature engineering. Such classification algorithms perform reasonably well in the discrimination between activities such as “wheelchair propulsion” and “non-wheelchair propulsion” of longer duration [46]. However, their performance is dissatisfactory in the classification of more complex single activities such as the SL-ADL of interest in this study. Deep learning techniques can automatically learn dynamically changing features from the raw sensor data [57]. Using the Mathworks deep learning toolbox, a deep learning model was constructed; its architecture is tabularized in Table 2. Most of the functions within this toolbox rely on the work of Bishop 2006 [63]. In the first instance, all activations, parameters, learning rates, and regularizations were kept as the default values of the toolbox.

Data from all five sensors at 10 Hz were used as the input (3D acceleration and 3D angular velocity for each sensor, and two channels of rectified smooth EMG), concatenated for all activities per participant, and for all but one participant while using a leaving one subject out approach (LOSO), as described in Section 2.6. The annotated activities from the video recordings served as the target for the training of the algorithms. 

### 2.6. Data Organization and Performance Measures

To train a generalizable algorithm, a LOSO approach was followed. This means that the training dataset consisted of data from nine participants, while data from the tenth participant served as the validation data; this procedure was repeated 10 times, each time using the data from another participant as the validation dataset. To prevent overfitting, early stopping was applied by monitoring the accuracy of the prediction of the validation dataset over the iterations in training. When the validation accuracy, after an initial 15 iterations, did not improve or even decreased over two consecutive iterations, training was stopped. As random weights are assigned to the connections between cells during the initialization of neural networks, this might lead to different results when repeated on exactly the same dataset. To account for such effects, the whole training procedure was repeated five times per participant; based on the prediction accuracy over all activities, the best performing network per participant was saved. 

**Figure 3:**
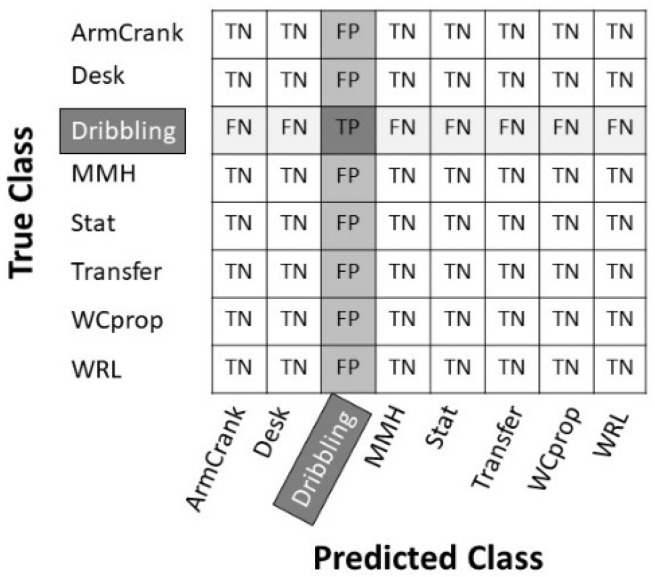
The multiclass confusion chart for class “Dribbling”.

From the resulting multiclass-confusion charts, the performance measures of the accuracy, sensitivity, precision, and specificity can be calculated according Equations (1)–(4). These performance measures are calculated per class, based on the values of the true positives (TP), true negatives (TN), false positives (FP), and false negatives (FN) for that class. See Figure 3 for an indication of these TP, TN, FP, and FN for the activity “Dribbling”.

(1) $$Accuracy=\frac{TP+TN}{TP+TN+FP+FN}$$

(2) $$Sensitivity=\frac{TP}{TP+FN}$$

(3) $$Precision=\frac{TP}{TP+FP}$$

(4) $$Specificity=\frac{TN}{TN+FN}$$

As a consecutive step, the amount of input data used to train the algorithms was reduced based on pragmatic considerations by reducing the number of sensors used to minimize the participant load for future measurements. The architecture of the algorithm and the LOSO approach remained unchanged, but was repeated for four different sensor combinations. Combination 1 used accelerometer and gyroscope signals from all five sensors, and two channels of EMG; combination 2 omitted the EMG, combinations 3 and 4 used only the accelerometer and gyroscope signals from either the upper arm and forearm sensor, respectively.

## 3. Results

**Figure 4:**
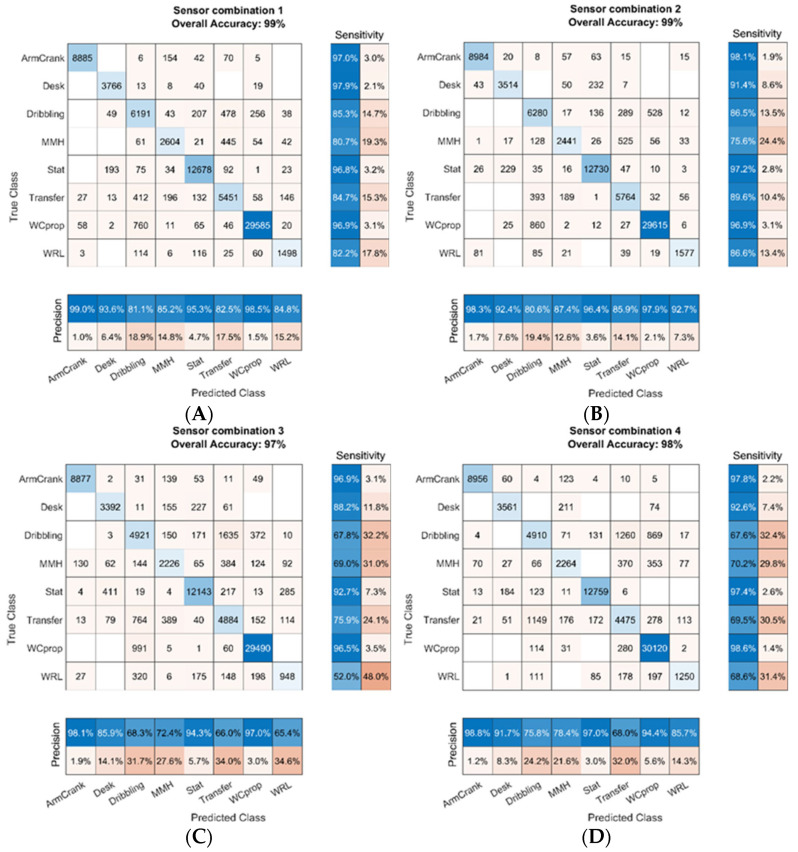
The confusion charts for the four sensor combinations, (**A**) = combination 1, (**B**) = combination 2, (**C**) = combination 3, (**D**) = combination 4.

Overall, the trained deep learning algorithm performed well in the classification of the relevant SL-ADL for data from the participant not used for training. The confusion charts in Figure 4 depict the performance over the LOSO approach for ten participants for the four sensor combinations. Each cell displays the number of datapoints for a given true and predicted class. Row-normalized values on the right of each chart depict the percentage of correctly classified data points for a given activity, which equals sensitivity. Column-normalized values below each chart depict the percentage of correctly classified datapoints for a given prediction, which equals precision.

The performance measures are summarized in Table 3, as derived from the confusion chart data in Figure 4, averaged over all of the participants and activities. Overall, the accuracy of the deep learning model reached values over 98%, indicating that 98% of the samples from the dataset were classified correctly. The omission of EMG (sensor combination 2) had no negative effect on the performance of the trained algorithms. When comparing setups that use data from a single sensor only, using forearm sensor data leads to a slightly higher sensitivity and precision than when using upper arm data, but both combinations performed worse than combination 1 or combination 2.

**Figure 5:**
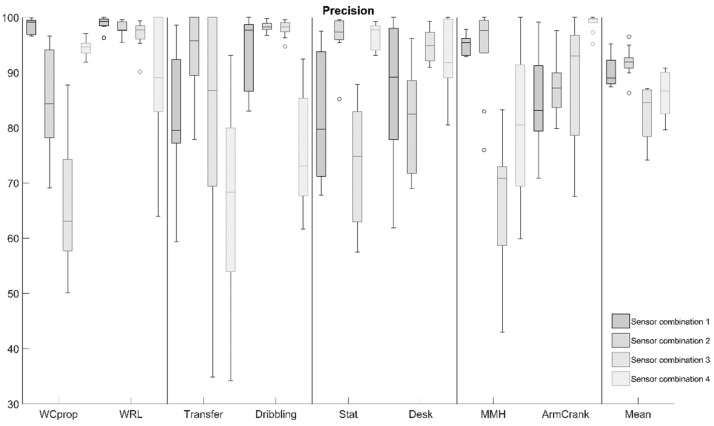
The box plots for “precision” of the four sensor combinations, indicating the median, upper, and lower quartiles (the box), minimum and maximum values (error bars) and eventual outliers (°).

**Figure 6:**
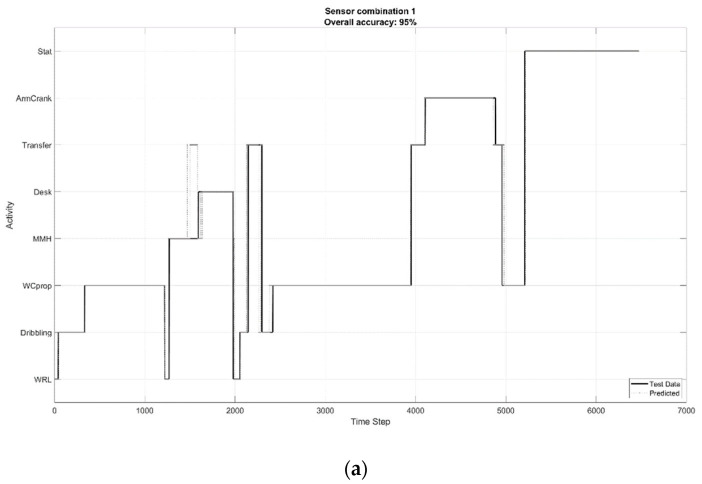

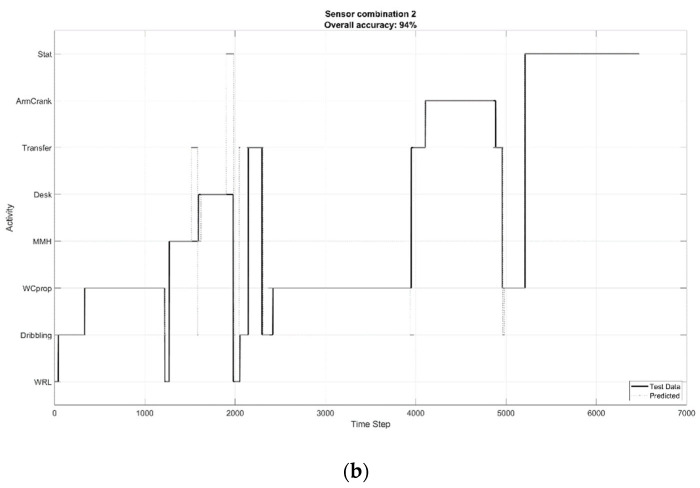

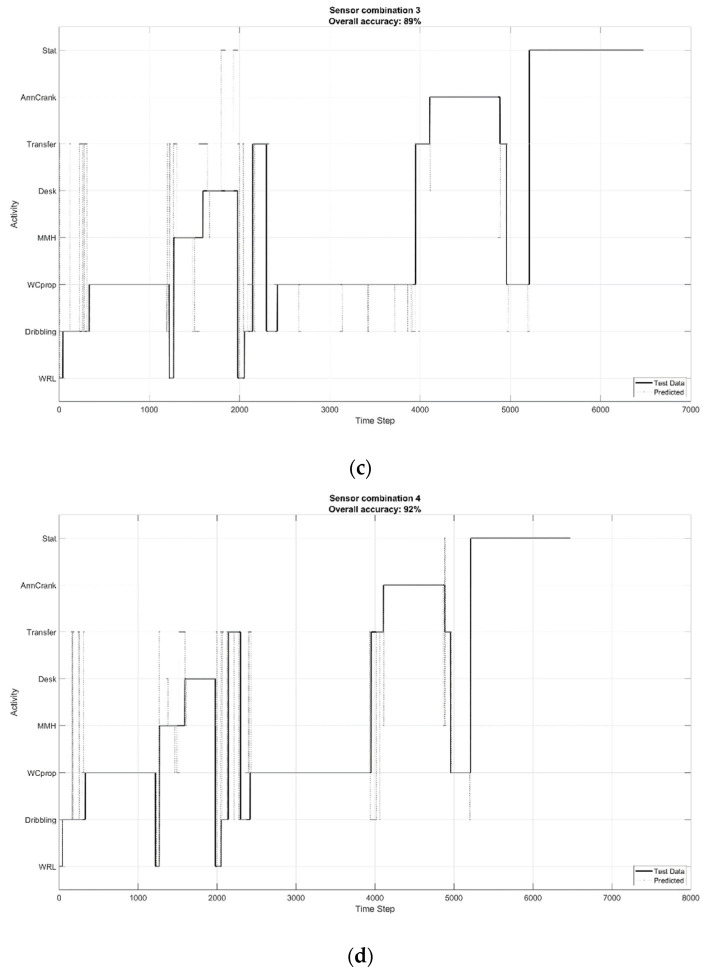
The time lines of predicted (dashed lines) versus test data (solid line) for one participant, for sensor combinations 1–4, indicated by (**a**–**d**) respectively).

The boxplots in Figure 5 depict the “precision” of the algorithms for the list of SL-ADL for the four sensor combinations, calculated over the ten participants. There was no setup that consistently shows a higher precision for all of the activities. For instance, with sensor combination 1, activities such as WCprop, WRL, and MMH were classified extremely well with little variation over participants, whereas arm cranking was best classified with sensor combination 4. When minimizing the number of sensors, an increase in the variation in the performance measures over participants could be observed, meaning that the method might work well for some participants, but not perform adequately for others. In Figure 6, the timelines of classification for one participant are visualized for the four sensor combinations. From these timelines, the behavior of the trained algorithms could easily be observed in terms of the frequency and duration of mismatches in classification when minimizing the input to data from one sensor. 

## 4. Discussion

The overall objective of the current study was to develop and validate a generalizable methodology for the classification of wheelchair related SL-ADL based on the wearable sensor data. In general, the trained deep learning algorithms showed an accuracy of over 98% in the classification of the selected SL-ADL, which opens the way for using such methodology in further research on shoulder loading behavior in MWU.

The combination of a GRU layer and a biLSTM layer appeared to be capable in learning the dynamic features of the sensor signals and thereby discriminating between the single SL-ADL of interest. This is an interesting step forward when compared to the results in literature, where the majority of studies have focused on the distinction between active wheelchair propulsion, passive wheelchair ambulation, and non-propulsive activity [45,46,47,56], with a variety of machine-learning algorithms. Despite accuracies of over 85% to 90%, these approaches did miss the level of detail required for the monitoring of SL-ADL at the MWU shoulder. The same accounts for a comparison to the state-of-the-art methods from the deep learning research community. The majority of publications have focused on gross whole-body movements, general activity classification for able bodied persons, for smartphone and smartwatch collected data, or on video based human activity recognition [66]. When compared to studies focusing on wheelchair related activities comparable to the relevant SL-ADL, we found that the obtained overall accuracy of 95–98% in this study was higher than the performance of classifiers such as linear or quadratic discriminant analysis, or support vector machines, as examined by Garciá-Massó et al. [43]. Other research groups addressed distinct activities from the perspective of physical activity monitoring, used data measured at activities with a duration of several minutes and extracted features over one-minute intervals [45]. Despite accuracies of over 88%, such an approach can only give information on the total duration of a certain activity over a day, but not, for instance, the number of transfers a day, which could be an important handle for interventions when addressing shoulder load.

The current study was embedded in a larger project, and a redundant set of variables was collected including the EMG of the biceps and medial deltoid. These two muscles were active during most of the included SL-ADL (based on visual inspection of the signals and experience) and were measured as a proxy for exerted force. The information on the activity of these two muscles is likely to have less discriminative power in the classification of SL-ADL, in comparison to the other signals collected. When reducing the number of sensors to be used to train the algorithm, it became clear that for the classification of the activities, the two channels of EMG were not required (combination 2), as the performance measures stayed about the same, or even improved for several activities. Such a reduction in the required equipment is a clear advantage in the practical feasibility of the method.

One of the motivations to use sensors is the search for a non-invasive and unobtrusive data collection of MWU in real-life settings. Camera and marker-based motion capture systems have the limitation of a fixed, stationary measurement volume, rendering them useless for long-term real-life measurements. Marker-less motion capture based on a camera attached to the wheelchair has quite an obtrusive appearance, and suffers from problems such as the occlusion of relevant body parts.

As it was not clear whether the specific SL-ADL of interest could be classified based on data from one sensor only (e.g., a smartwatch), a redundant sensor setup was applied. This redundancy on the number of sensors required for classification of the SL-ADL was reduced by post hoc analysis. The Shimmer IMUs are not the smallest available; however, the concurrent measurement of EMG is an initial requirement. Since the collection of EMG does not seem to be required, a further reduction in sensor size can be considered.

### 4.1. Future Research

When minimizing the input for training the algorithms to data from one single sensor (upper arm or forearm), the performance of the trained classification algorithms was more diffuse. These algorithms performed better in the classification of some SL-ADL, but much worse in the classification of others, or showing more variation in performance over participants. A solution here might be leaving the LOSO approach, which aims at training a generalizable algorithm, and train an algorithm on an individual’s dataset. This would mean that for a successful training of an SL-ADL monitor, initially, the SL-ADL of interest should be measured from that specific individual, and used for the training of an algorithm. Potentially, a more complex architecture of the algorithm (e.g., with multiple GRU and biLSTM layers) might also lead to a more consistent performance over the participants, at the cost of a higher computational load when training such algorithms.

### 4.2. Limitations

To examine the feasibility of machine learning in classifying wheelchair related SL-ADL from wearable sensors, able bodied participants were trained in the SL-ADL of interest. It is expected that this methodology will perform equally well on data collected from experienced wheelchair users. This, however, has to be examined before being used as a tool for further research. Additionally, current proof of principle is based on single instructed activities, executed in a laboratory environment. As the execution of these SL-ADL in real-life is likely to be much more variable, in terms of execution times, frequency, and also by fluent combination with other activities (e.g., dribbling while handling materials), it is very well possible that the current algorithms are not performing satisfactorily in classifying data from real-life settings. An obvious solution would be training such algorithms on labeled data from real-life settings, which is our next step in research. Last but not least, the current algorithms classify a list of eight distinct SL-ADL. However, any collected data will be classified as one of these eight SL-ADL, which is not realistic for data collected in real-life settings, where many other activities are executed. One option is to define a “remainder” category for those activities not of interest. Another option lies in combining unsupervised learning (self-organizing maps) with supervised learning (the currently developed and described method) to capture the features of all activities performed, but only classify the relevant ones.

## 5. Conclusions

A generalizable algorithm could be trained by a deep learning model to classify wheelchair related SL-ADL from wearable sensor data. EMG is not necessarily needed, but a setup with five sensors performed better than only one sensor. Using only one sensor slightly reduced the performance of the method, but increased the variability in performance over the participants. The proof of principle appears to be successful and despite the fact that several hurdles still have to be overcome, the approach opens the way toward real-life applications.

## Figures and Tables

**Table 1:** A list of addressed SL-ADL and their description, used as instructions for the participants and as a definition for the annotation of the video recordings.

	SL-ADL	Abbreviation	Description
1	Weight Relief Lift	WRL	Weight relief lift, starts with placing hands on the rim of the wheel, then push up, hold, and release to sit. Activity stops when hands start moving away from the rim.
2	Dribbling	Dribbling	Intermitted wheelchair propulsion in restricted space (maximal 3 m distance covered), maximal 3 pushes including turns and backward propulsion. Starts with first rotation of the wheel, ends when wheel stops rotating.
3	Wheelchair propulsion	WCprop	Continuous wheelchair propulsion on the treadmill at 0.56 and 1.11 m/s at 0%, and 0.56 m/s at 6% inclination.
4	Manual material handling	MMH	Pick and place a weight of 2 kg to four individual shelves from a cupboard. Starts from rest, as the hand starts moving to pick up the weight for the first time, until release of the weight after completing the sequence.
5	Deskwork	Desk	Sitting at desk, typing on a key board, using the mouse and mobile phone.
6	Stationary	Stat	Sitting still in wheelchair, some movement of the hands allowed (adjusting hair, repositioning hands, gestures while chatting, etc.).
7	Transfer	Transfer	Transfer from wheelchair to couch or vice versa. Transfer starts when reaching out with the hands to the next object to transfer to, until sitting on that object. Repositioning before and after transfer is considered WRL.
8	Arm Cranking	ArmCrank	Arm crank ergometer work at 60 rpm.

**Table 2:** The architecture, layers, and parameters of the deep learning model used.

Layer	Function	Parameters
1. Sequence input	Read data as sequences	Neither normalization nor centering or scaling was applied
2. Gated recurrent unit (GRU)	Recurrent network with gated units that solves vanishing/exploding gradient problems, as introduced by Cho et al. 2014 [64]	100 hidden units
3. Bidirectional Long Short Term Memory layer (biLSTM)	Special mode of recurrent neural networks to learn long-term dependencies, developed by Hochreiter and Schmidhuber 1997 [65]	200 units
4. Fully connected layer	Takes the output of the multiplies the output of the biLSTM with a weight matrix and adds a bias vector	Output size 8 classes
5. Softmax layer	Applies a softmax function to the input, usually followed by a classification layer for classification problems	Default values used
6. Classification layer	A classification layer computes the cross-entropy loss for classification and weighted classification tasks with mutually exclusive classes	Default values used

**Table 3:** Performance measures (in %, mean (SD)) of the trained algorithms for the four different sensor combinations.

Sensor Combination	Accuracy	Sensitivity	Precision	Specificity
1: 5 IMUs + 2 EMG	98.4 (1.31)	89.8 (9.62)	90.2 (10.40)	99.1 (1.04)
2: 5 IMUs	98.5 (1.23)	90.1 (10.32)	91.9 (8.63)	99.1 (1.10)
3: 1 IMU on upper arm	97.2 (2.19)	79.1 (19.30)	82.4 (18.20)	98.3 (1.64)
4: 1 IMU on forearm	97.6 (2.25)	82.2 (18.36)	86.1 (16.11)	98.5 (1.75)

## Data Availability

The data presented in this study are available on request from the corresponding author.
